# Direct Comparison of Virtual-Histology Intravascular Ultrasound and Optical Coherence Tomography Imaging for Identification of Thin-Cap Fibroatheroma

**DOI:** 10.1161/CIRCIMAGING.115.003487

**Published:** 2015-10-20

**Authors:** Adam J. Brown, Daniel R. Obaid, Charis Costopoulos, Richard A. Parker, Patrick A. Calvert, Zhongzhao Teng, Stephen P. Hoole, Nick E.J. West, Martin Goddard, Martin R. Bennett

**Affiliations:** From the Division of Cardiovascular Medicine (A.J.B., D.R.O., C.C., P.A.C., M.R.B.), Department of Radiology (Z.T.), and Department of Engineering (Z.T.), University of Cambridge, Cambridge, United Kingdom; Health Services Research Unit, Usher Institute of Population Health Sciences and Informatics, College of Medicine and Veterinary Medicine. University of Edinburgh, Edinburgh, United Kingdom (R.A.P.); and Departments of Interventional Cardiology (S.P.H., N.E.J.W.) and Pathology (M.G.), Papworth Hospital NHS Trust, Cambridge, United Kingdom.

**Keywords:** atherosclerosis, autopsy, coronary artery disease, lipids, myocardial infarction

## Abstract

Although rupture of thin-cap fibroatheroma (TCFA) underlies most myocardial infarctions, reliable TCFA identification remains challenging. Virtual-histology intravascular ultrasound (VH-IVUS) and optical coherence tomography (OCT) can assess tissue composition and classify plaques. However, direct comparisons between VH-IVUS and OCT are lacking and it remains unknown whether combining these modalities improves TCFA identification.

**Methods and Results—:**

Two hundred fifty-eight regions-of-interest were obtained from autopsied human hearts, with plaque composition and classification assessed by histology and compared with coregistered ex vivo VH-IVUS and OCT. Sixty-seven regions-of-interest were classified as fibroatheroma on histology, with 22 meeting criteria for TCFA. On VH-IVUS, plaque (10.91±4.82 versus 8.42±4.57 mm^2^; *P*=0.01) and necrotic core areas (1.59±0.99 versus 1.03±0.85 mm^2^; *P*=0.02) were increased in TCFA versus other fibroatheroma. On OCT, although minimal fibrous cap thickness was similar (71.8±44.1 μm versus 72.6±32.4; *P*=0.30), the number of continuous frames with fibrous cap thickness ≤85 μm was higher in TCFA (6.5 [1.75–11.0] versus 2.0 [0.0–7.0]; *P*=0.03). Maximum lipid arc on OCT was an excellent discriminator of fibroatheroma (area under the curve, 0.92; 95% confidence interval, 0.87–0.97) and TCFA (area under the curve, 0.86; 95% confidence interval, 0.81–0.92), with lipid arc ≥80° the optimal cut-off value. Using existing criteria, the sensitivity, specificity, and diagnostic accuracy for TCFA identification was 63.6%, 78.1%, and 76.5% for VH-IVUS and 72.7%, 79.8%, and 79.0% for OCT. Combining VH-defined fibroatheroma and fibrous cap thickness ≤85 μm over 3 continuous frames improved TCFA identification, with diagnostic accuracy of 89.0%.

**Conclusions—:**

Both VH-IVUS and OCT can reliably identify TCFA, although OCT accuracy may be improved using lipid arc ≥80° and fibrous cap thickness ≤85 μm over 3 continuous frames. Combined VH-IVUS/OCT imaging markedly improved TCFA identification.

## Abstract 2

Supplemental Digital Content is available in the text.

Autopsy studies have demonstrated that around two thirds of all myocardial infarctions are because of plaque rupture, resulting in luminal thrombosis.^1^ Ruptured plaques are characterized by a large, lipid-rich necrotic core (NC) with an overlying, thin fibrous cap.^2^ A precursor lesion for rupture, termed a thin-cap fibroatheroma (TCFA), displays similar plaque compositional features to ruptured plaques. As such, imaging modalities that can identify TCFAs before rupture are of considerable clinical importance.

**See Clinical Perspective**

**See Editorial by Mintz**

Virtual-histology intravascular ultrasound (VH-IVUS) is an invasive imaging modality that uses radiofrequency ultrasound backscatter data to identify plaque components, including NC, calcification, fibrous, and fibrofatty tissue. In ex vivo studies, VH-IVUS has predictive accuracies of >93.5% to characterize coronary plaque composition^3^ and a diagnostic accuracy of 76% for TCFA.^4^ Furthermore, despite VH-IVUS not having the resolution to measure a thin fibrous cap, multiple prospective studies demonstrate that VH-IVUS–defined TCFA (VH-TCFA) are associated with future major adverse cardiovascular events,^5–7^ confirming their biological importance. Nevertheless, overall event rates attributable to each high-risk plaque in these studies were low (<10%) and the prevalence of VH-TCFA in vivo far exceeded that observed in autopsy studies,^8^ suggesting that VH-IVUS may overestimate TCFA prevalence.

Intravascular optical coherence tomography (OCT) is an alternative technique that allows plaque characterization using near-infrared light to display high-resolution (≈20 μm) images of coronary lesions. Validation studies have suggested that OCT has sensitivities around 75% for fibrous, 95% for fibrocalcific, and 92% for lipid-rich plaques.^9^ The high resolution of OCT also has potential to identify plaque features not visible on VH-IVUS, including measurement of fibrous cap thickness (FCT).^10^ Although prospective studies are lacking, lipid-rich plaques on OCT are more commonly associated with acute coronary syndromes,^11^ and OCT-defined TCFA are more numerous in angiographically mild lesions, an observation consistent with autopsy data.^12^

Although clinical studies have used VH-IVUS and OCT to describe coronary atherosclerosis, their ability to identify TCFA and discriminate TCFA from other fibroatheroma have never been directly compared. We assessed VH-IVUS and OCT characteristics of advanced lesions, as defined by histology, and identified features of each imaging modality that optimally discriminate between TCFA and other fibroatheroma. We next tested the ability of both VH-IVUS and OCT to correctly identify TCFA using existing plaque classification definitions and assessed whether refined imaging cut-off values could improve TCFA identification. Finally, we assessed whether combined VH-IVUS/OCT was more accurate for TCFA identification than either modality alone.

## Methods

### Ex Vivo Imaging

The study protocol was approved by the Cambridgeshire Research and Ethics Committee (Ref. 07/H0306/123) and consent obtained from relatives. Arteries were harvested from human hearts during autopsy in consultation with a senior pathologist. Hearts were excluded if coronary artery thrombosis was the suspected cause of death. All vessels were stored immediately in phosphate-buffered saline at 4°C and imaged within 48 hours of death. The left anterior descending artery (n=14) was dissected and excised, including ≈40 mm of surrounding tissue to maintain overall structural integrity.^4^ Side branches were ligated and a guide catheter sutured into the left coronary ostium. A 0.014″ coronary guidewire (either BMW Universal or Pilot 50, Abbott Vascular) was advanced permitting delivery of intravascular catheters. The vessel was fixed to a proprietary designed rig and prewarmed (to 37°C). Vessels were imaged under pressure–perfusion at 100 mm Hg before histological processing. VH-IVUS data were acquired using 20-MHz Eagle-Eye Gold catheters (Volcano Corporation) at 0.5 mm/s pullback, with radiofrequency data being captured on the R wave provided by an ECG signal generator. OCT data were acquired by DragonFly C7 catheters (St. Jude Medical) using 25.0 mm/s automated pullback. All imaging data were digitally stored and exported for offline analysis.

### Histological Processing

After imaging, arteries were fixed in 10% buffered formalin for ≥24 hours. Five millimeter longitudinal intervals were marked along the length of the artery as regions-of-interest (ROI). Vessels underwent decalcification if necessary using EDTA followed by histological processing. Histological sections of 5-μm thickness were then cut at 400-μm intervals throughout ROI, maintaining proximal and distal orientation. Histological sections were stained with hematoxylin-eosin and Van Gieson and reviewed by an independent, experienced cardiac pathologist, blinded to all intracoronary imaging. Reading of the histological sections and imaging data was performed in a random order. Plaque classification was performed based on the modified AHA classification scheme^2^ as follows. Adaptive intimal thickening was plaque with evidence of fibrous tissue but without lipid pools; pathological intimal thickening had evidence of extracellular lipid accumulation but without a defined NC; fibrocalcific plaques were plaques with large areas of calcification with small, or absent, NCs, and fibroatheroma were plaques containing necrotic lipid core with inflammatory cellular infiltration. A TCFA was defined as any fibroatheroma with an FCT <65 μm.

### Virtual-Histology Intravascular Ultrasound Analysis

**Figure 1.**
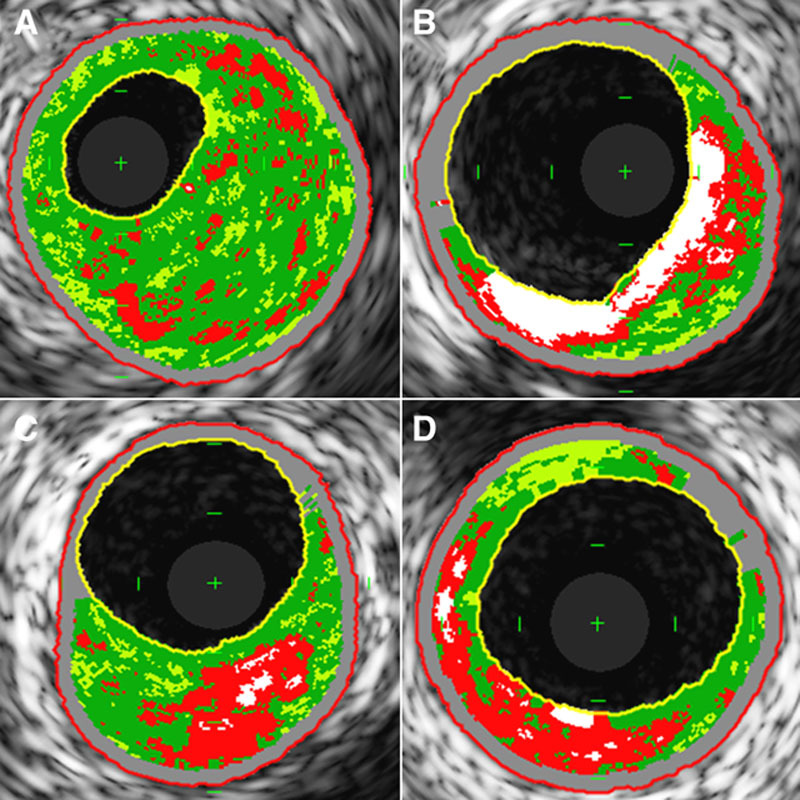
Plaque classification using virtual-histology intravascular ultrasound. **A**–**D**, Examples of 4 virtual-histology intravascular ultrasound plaques (plaque burden >40%), classified as pathological intimal thickening (**A**), fibrocalcific plaque (**B**), thick-cap fibroatheroma (**C**), and thin-cap fibroatheroma (**D**).

VH-IVUS data were analyzed offline using echoPlaque 4.0 (Indec Medical Systems) by 2 independent observers blinded to histology and OCT. Both luminal and external elastic membrane borders were manually corrected for each frame, permitting measurement of gray-scale IVUS features, including plaque burden and luminal area. Once borders were defined, the backscattered ultrasound data were automatically processed, with colors assigned for each tissue component: fibrous (dark green), fibrofatty (light green), NC (red), and dense calcium (white). VH-defined plaque classification (Figure 1) was performed using an established classification system; full details are found in the Methods section of Data Supplement.^6^ A consensus was obtained in cases of disagreement and this value used in subsequent analysis. Interobserver agreement for VH-defined plaque classification was strong (κ=0.84; 95% confidence interval [CI], 0.78–0.90; Table I in the Data Supplement).

### OCT Analysis

**Figure 2.**
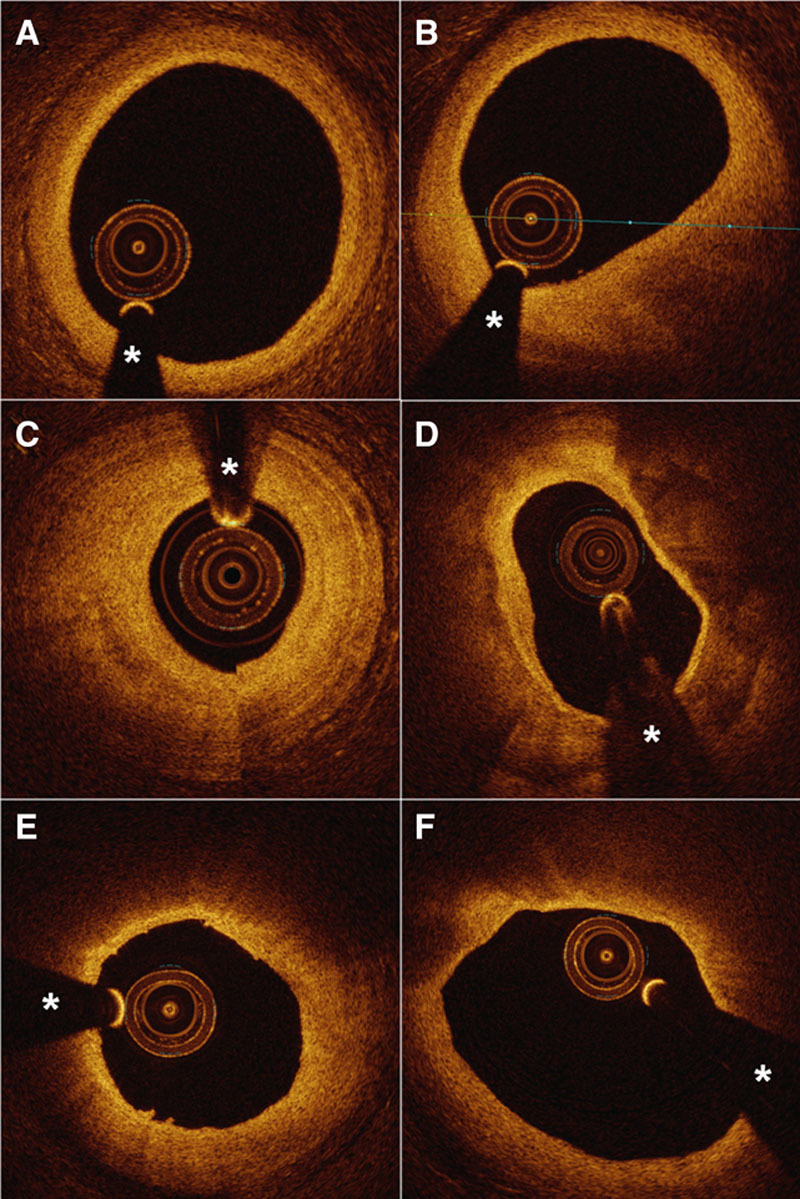
Plaque classification using optical coherence tomography. Optical coherence tomographic images illustrating both nonatherosclerotic vessel (**A**) and plaque (**B**), with evidence of focal intimal thickening and loss of layered vessel wall structure in **(B**). **C**–**F**, Further examples of fibrous plaque (**C**), fibrocalcific plaque (**D**), thick-cap fibroatheroma (**E**), and thin-cap fibroatheroma (**F**). Coronary guidewire artifact is denoted by * in all images.

Offline OCT analysis was performed using LightLab Imaging workstation (St. Jude Medical) by 2 independent observers blinded to histology and IVUS. Luminal contours were manually adjusted and plaque composition assessed for each frame. Lipid was defined as a signal-poor region within a plaque, with poorly delineated borders and a fast drop-off in OCT signal, whereas calcium was defined as a signal-poor region, but with sharply delineated borders and a gradual drop-off in OCT signal.^10^ Lipid arc (LA) measurements were recorded within each frame and mean (LA_mean_) and maximum (LA_max_) value calculated. If lipid was present in a frame, minimal FCT (FCT_min_) was measured at its thinnest part 3× times and mean value used in subsequent analysis.^12^ The number of continuous OCT frames with FCT_min_ ≤85 μm was also documented. OCT Plaque classification was performed according to an international consensus statement (Figure 2)^10^; an OCT-defined fibroatheroma was defined as plaque with LA_max_ ≥90°, with an OCT-defined TCFA having an FCT_min_ ≤85 μm, a value ≈20% higher than the histological definition to account for tissue shrinkage.^13^ Full details with illustrations of OCT plaque classification can be found in the Methods section of Data Supplement. Interobserver agreement for OCT plaque classification was strong (κ=0.89; 95% CI, 0.83–0.94; Table II in the Data Supplement).

### Statistical Analysis

Continuous data are expressed as mean±SD, whereas categorical data are presented as counts (or percentages). Agreement between VH-IVUS and OCT plaque classification observers was quantified by Cohen κ test. Plaque composition between lesion subtypes was compared using a nested ANOVA or *t* test where appropriate, as changes in plaque morphology may also be affected by differences in individuals, rather than differences in plaque morphology. For this, a hierarchical tree was created, first nesting each ROI within an atherosclerotic plaque and second with the donor heart itself, as each heart only provided 1 artery for the analysis. To assess diagnostic ability, the sensitivity, specificity, positive predictive value, negative predictive value, and diagnostic accuracy for each imaging modality were calculated, with corresponding Clopper–Pearson 95% CIs. These CIs were adjusted for clustering in the data by dividing the sample size by a design effect (which was computed as a function of the average number of ROI in each artery and the intracluster correlation coefficient) to obtain an effective sample size on which intervals were based. Receiver-operating characteristic curves were calculated by plotting sensitivity versus (1 -specificity), allowing calculation of area under the curve (AUC). Statistical analyses were performed both in SPSS 21.0.0 (SPSS Inc, IBM Computing) and R 2.10.1 (The R Foundation for Statistical Computing).

## Results

### Clinical Demographics

Fourteen human hearts were harvested and imaged. Demographics of donors are presented in Table 1. Donors ranged in age from 47 to 85 years, with 71.4% being male. As atherosclerosis is almost uniformly present in this age group, hearts were obtained from donors experiencing both cardiovascular death and noncardiovascular death, although death because of presumed coronary thrombosis was excluded.

**Table 1.**
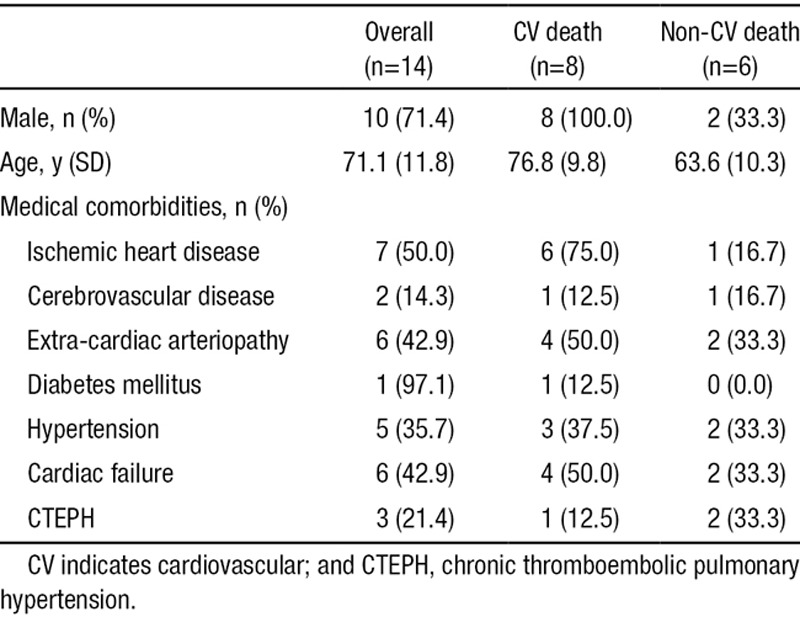
Clinical Characteristics of Donors for Autopsy Studies

### ROI Features and Composition

**Figure 3.**
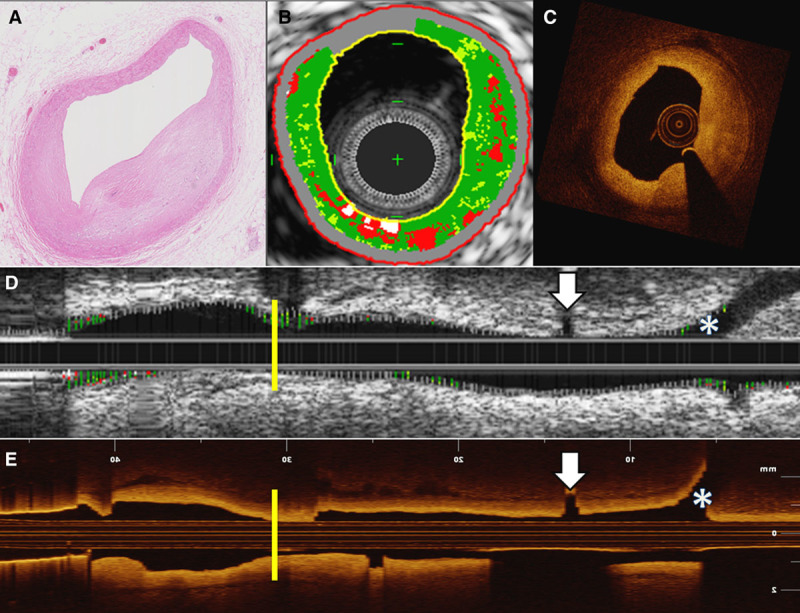
Coregistration between histology and intravascular imaging. Example of a plaque classified as pathological intimal thickening on histology (**A**), with coregistered virtual-histology intravascular ultrasound (VH-IVUS; **B**) and optical coherence tomography (OCT; **C**) images. Corresponding longitudinal VH-IVUS (**D**) and OCT (**E**) pullbacks denoting the location of the plaque within these vessels (yellow bar), with a small side-branch (arrow) and large bifurcation (*) used to assist colocalization.

Overall, 1290 mm of coronary artery were analyzed representing 258 histological ROI, with VH-IVUS available for 211 (81.8%), OCT for 207 (80.2%), and dual imaging for 200 (77.5%). VH-IVUS and OCT image sets were matched to coregistered histological ROI by an experienced intravascular imaging investigator, blinded to final histological plaque classification. Coregistration was aided by detailed measurements taken during ex vivo imaging using fiduciary landmarks, including bifurcations, guide catheter location, and prominent calcific deposits (Figure 3). By histology, ROI were classified as adaptive intimal thickening 97 (37.6%), pathological intimal thickening 50 (19.4%), fibrocalcific 38 (14.7%), and fibroatheroma 73 (28.3%). Of the ROI classified as fibroatheroma, 22 met the criteria for TCFA (8.5% of total ROI) with mean FCT on histology being 43.0±16.8 μm. All TCFA were imaged by both VH-IVUS and OCT. ROI that could not be imaged were all located in distal coronary segments and were classified as adaptive intimal thickening (66.7%), pathological intimal thickening (17.5%), fibrocalcific (8.7%), and fibroatheroma (7.0%).

We analyzed changes in plaque composition on ROI according to histological classification with increasing lesion complexity, focusing particularly on features that differ between histological TCFA and other fibroatheroma (Tables 2 and 3). Plaque area and burden on gray-scale IVUS increased significantly (*P*<0.001 using nested ANOVA) as plaques progressed, associated with increasing areas of fibrous, fibrofatty, NC, and dense calcium (*P*<0.001 using nested ANOVA) on VH-IVUS. Plaque area was significantly increased in TCFA compared with other fibroatheroma on gray-scale IVUS (10.91±4.82 versus 8.42±4.57 mm^2^; *P*=0.01), although plaque burden was similar (60.4±8.9 versus 58.6±12.0%; *P*=0.10). Compared with other fibroatheroma, TCFA had increased areas of fibrous tissue (3.83±1.99 versus 3.01±2.12 mm^2^; *P*=0.03), NC (1.59±0.99 versus 1.03±0.85 mm^2^; *P*=0.015), dense calcium (1.22±1.31 versus 0.65±0.79 mm^2^; *P*=0.03), and NC % (20.8±5.1 versus 16.0±7.3%; *P*=0.049) on VH-IVUS.

**Table 2.**
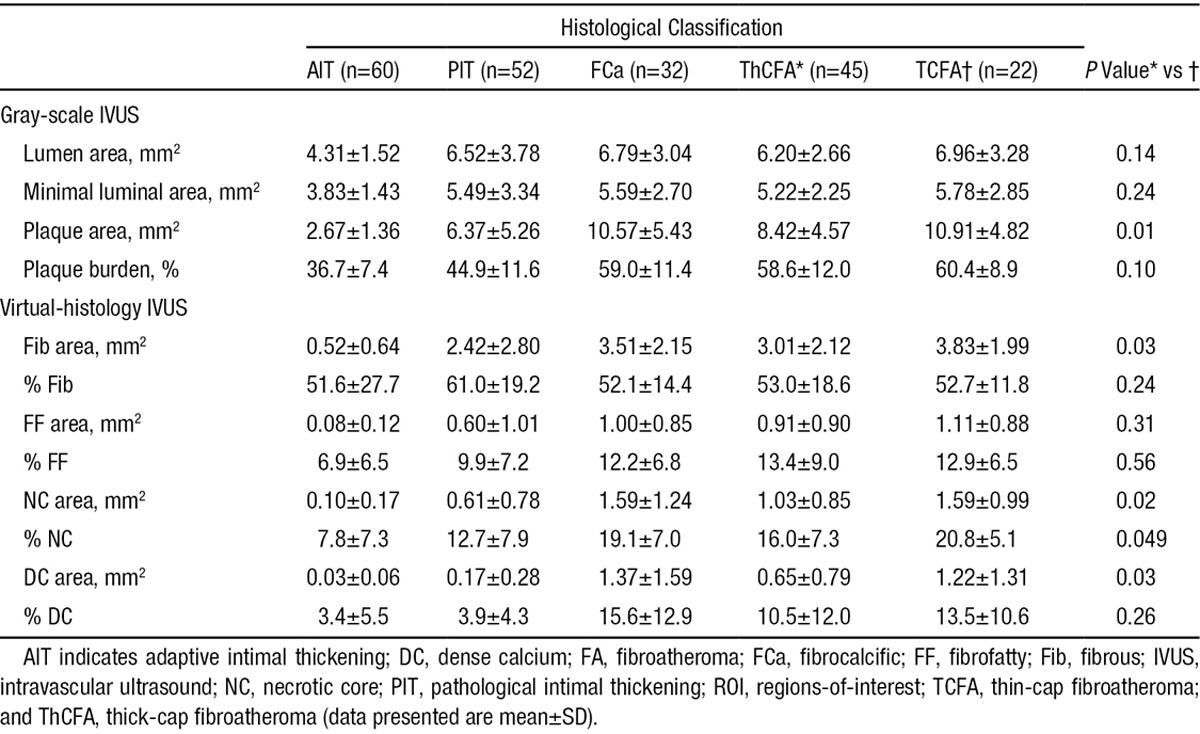
Virtual-Histology Intravascular Ultrasound Features for Each Plaque Subtype (ROI Numbers are Given in Parentheses)

**Table 3.**
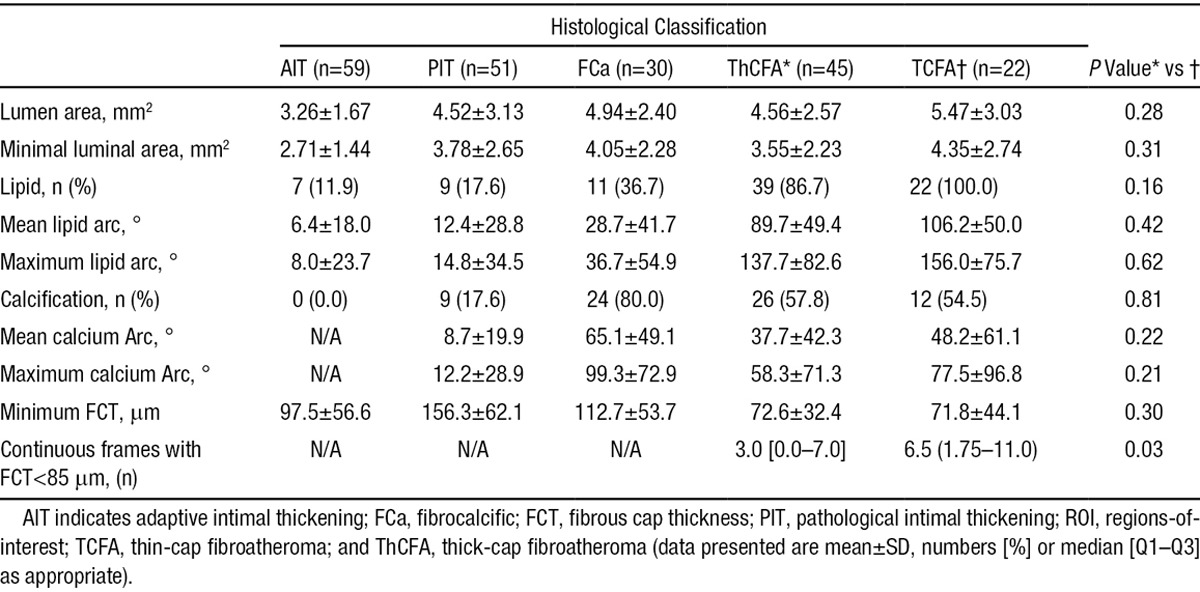
Optical Coherence Tomographic Features for Each Plaque Subtype (ROI Numbers are Given in Parentheses)

OCT-defined measures of atherosclerotic plaque composition also increased with increased stages of plaque progression, as defined by histology. There was a significant increase in % of plaques containing lipid (eg, adaptive intimal thickening 11.9% versus TCFA 100.0%; *P*<0.001) and for maximum LA (LA_max_ eg, adaptive intimal thickening 8.0±23.7 versus TCFA 156.0±75.7°; *P*<0.001). FCT_min_ reduced as lesions progressed (eg, pathological intimal thickening 156.3±62.1 versus TCFA 71.8±44.1 μm; *P*<0.001). Importantly, both LA_max_ (156.0±75.7 versus 137.7±82.6°; *P*=0.62) and FCT_min_ (71.8±44.1 versus 72.6±32.4 μm; *P*=0.30) were similar when TCFA and other fibroatheroma were compared; however, the number of frames of OCT with FCT<85 μm was significant higher in TCFA (6.5 [1.75–11.0] versus 2.0 [0.0–7.0]; *P*=0.03).

### Plaque Classification

**Figure 4.**
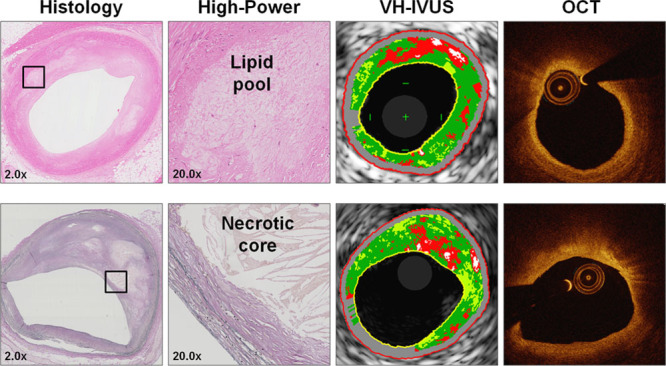
Examples of extracellular lipid accumulation and necrotic core correctly identified by intracoronary imaging. Examples of 2 plaques seen on histology that were correctly identified as containing extracellular lipid accumulations (**top**) and necrotic core (**bottom**) by both virtual-histology intravascular ultrasound (VH-IVUS) and optical coherence tomography (OCT). High power images (×20) are sampled from areas outlined in low power images (×2).

**Figure 5.**
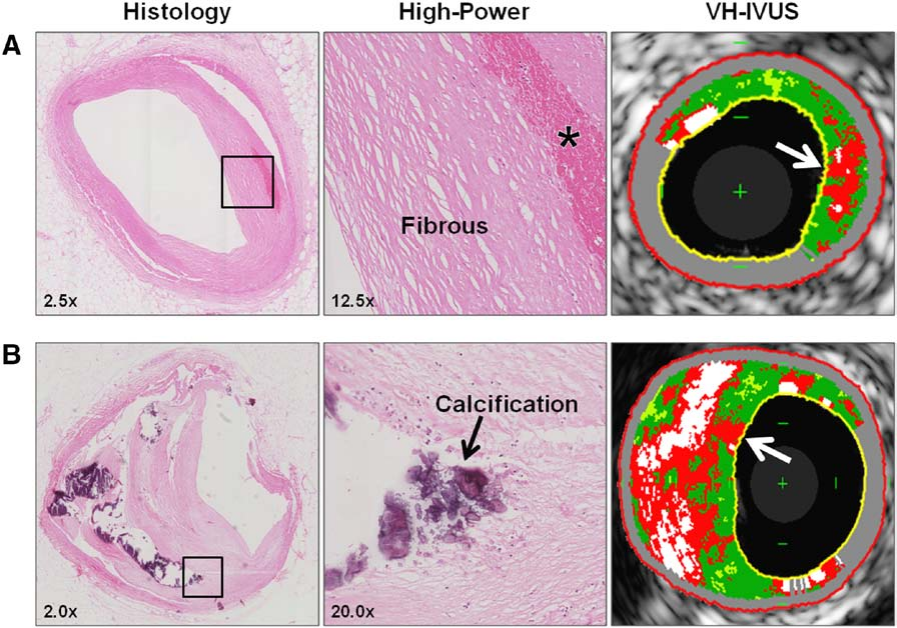
Examples of plaques incorrectly classified as virtual-histology thin-cap fibroatheroma (VH-TCFA). **A** and **B**, Examples of 2 coronary plaques incorrectly classified as TCFAs by VH-IVUS. Reasons for misclassification include incorrect tissue characterization (**A**), with an area of intraplaque hemorrhage (*) being depicted as necrotic core and artifactual regions of necrotic core adjacent to a large plate of calcification (**B**). For both plaques, confluent necrotic core was >10% and in contact with the luminal contour (arrows), resulting in VH-TCFA classification. High power images represent areas outlined on histology (**left**).

**Figure 6.**
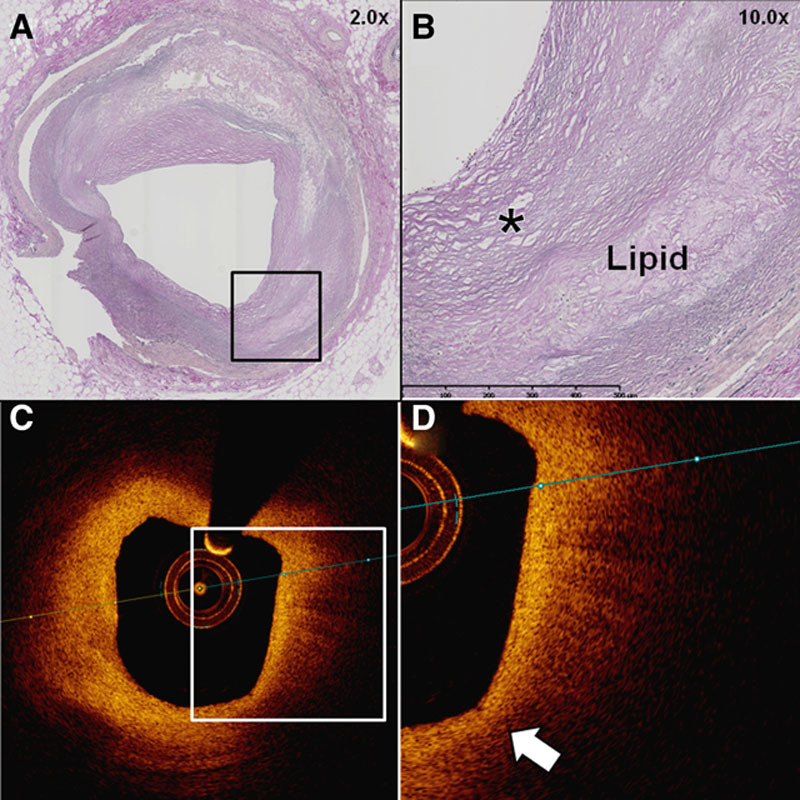
Example of plaque incorrectly classified as optical coherence tomography thin-cap fibroatheroma (OCT-TCFA). **A**–**D**, Histological image of a fibroatheroma (**A**) displaying evidence of extracellular lipid accumulation (**B**) and thick (>65 μm) overlying fibrous cap (*) with coregistered OCT image (**C**). The OCT displays a signal-poor region with poorly delineated borders (**D**) correctly identifying lipid, but the minimal fibrous cap thickness (measured at arrow) was <85 μm, resulting in classification as OCT-TCFA.

Although individual features may differ subtly between TCFA and other fibroatheroma on VH-IVUS and OCT, plaque classification uses a combination of imaging features to define plaque type as compared with standard histological definitions (Figures 1 and 2; Methods section of Data Supplement). Overall, the prevalence of VH-TCFA was 20.9% and for OCT-TCFA was 20.2%. The diagnostic accuracies for any fibroatheroma and TCFA were 77.5% and 76.5% for VH-IVUS and 89.0% and 79.0% for OCT, respectively (Figure 4; Table 4), with sensitivities to detect TCFA of 63.6% (VH-IVUS) and 72.7% (OCT). The incorrectly classified TCFA showed several common features. For example, 7 of 8 TCFA (87.5%) not identified by VH-IVUS were classified as thick-cap fibroatheroma, indicating that although VH-IVUS can identify large areas of NC, it has difficulty discriminating thin fibrous caps. In contrast, false-positive VH-TCFA identification was commonly encountered in regions of calcification, where the adjacent plaque composition was incorrectly portrayed as NC (Figure 5). Three of six TCFA incorrectly classified by OCT had an LA_max_ <90° and 3 of 6 had FCT_min_ ≥85 μm (Figure 6), suggesting that current thresholds for TCFA identification by OCT may not be accurate.

**Table 4.**
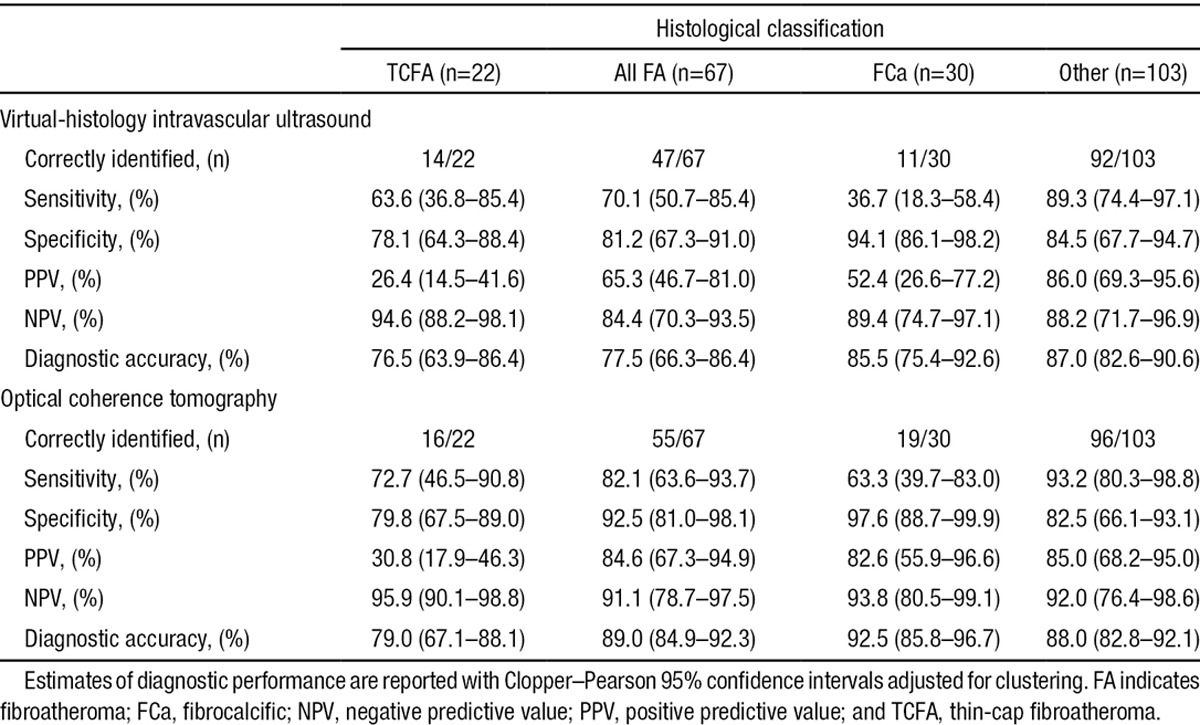
Accuracy of Virtual-Histology Intravascular Ultrasound and Optical Coherence Tomographic Plaque Classification Compared With Histology

### Assessment of Specific Imaging Features to Identify Advanced Plaques

**Figure 7.**
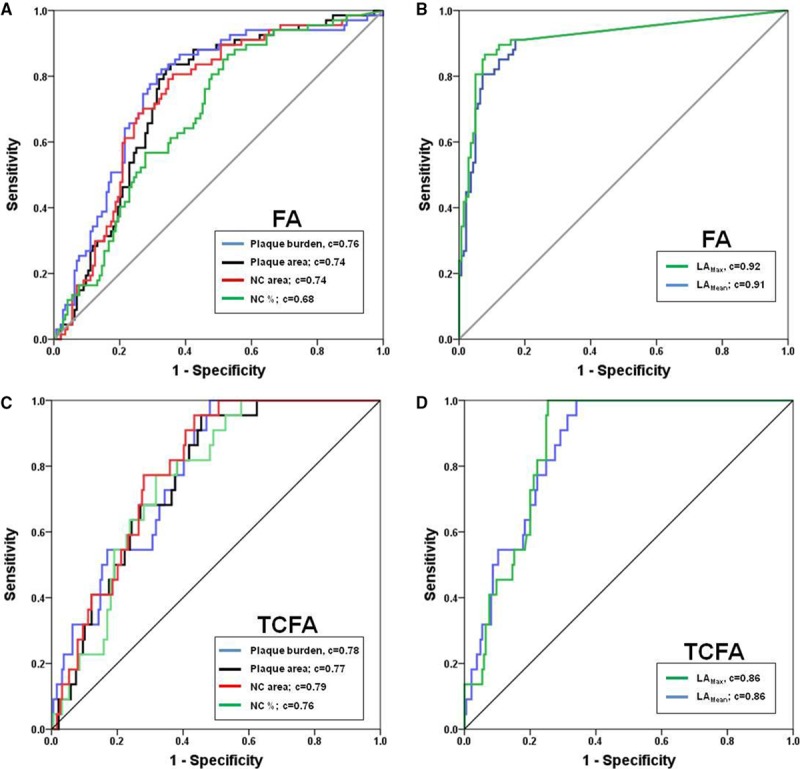
Receiver-operating curves for identification of advanced coronary plaques. **A**–**D**, Receiver-operating curves for the identification of all fibroatheroma (FA) and thin-cap fibroatheroma (TCFA) using virtual-histology intravascular ultrasound ((**A** and **C**) and OCT (**B** and **D**).

These results suggest that existing definitions for TCFA using VH-IVUS and OCT may lead to misclassification in ≈20% of plaques, and we need better indicators of TCFA. NC area and % and plaque area were increased in TCFA; we therefore assessed the ability of these features to identify any fibroatheroma or TCFA by receiver-operating characteristic analysis. Confluent NC area (AUC, 0.74; 95% CI, 0.67–0.81 and AUC, 0.79; 95% CI, 0.72–0.86) and NC percentage (AUC, 0.68; 95% CI, 0.61–0.76 and AUC, 0.76; 95% CI, 0.68–0.84) were moderate predictors of fibroatheroma and TCFA, respectively (Figure 7A and 7C). Plaque area (FA: AUC, 0.74; 95% CI, 0.67–0.80 and TCFA: AUC 0.77; 95% CI, 0.69–0.85) and plaque burden (FA: AUC, 0.76; 95% CI, 0.70–0.83 and TCFA: AUC, 0.78; 95% CI, 0.70–0.86) had similar predictive abilities to identify any fibroatheroma and TCFA (*P*>0.05 for all comparisons with NC area and %).

We next assessed the ability of OCT measures to identify any fibroatheroma or TCFA (Figure 7B and 7D). Both LA_mean_ and LA_max_ were excellent discriminators of fibroatheroma and TCFA from other lesions (fibroatheroma LA_mean_: AUC, 0.91; 95% CI, 0.86–0.96 and LA_max_: AUC, 0.92; 95% CI, 0.87–0.97 and TCFA LA_mean_: AUC, 0.86; 95% CI, 0.80–0.92 and LA_max_: AUC, 0.86; 95% CI, 0.81–0.92). The optimal LA_max_ cut-off value for fibroatheroma and TCFA identification was 80.0°, which gave sensitivity and specificity of 86.7% and 92.1% for fibroatheroma and 100.0% and 74.6% for TCFA. For FCT, the highest discriminatory power was observed using the number of continuous frames with FCT_min_ ≤85 μm (AUC, 0.83; 95% CI, 0.73–0.93).

### Combined Imaging for Identification of Advanced Plaques

Although both VH-IVUS and OCT can image coronary atherosclerosis, the modalities have different capabilities. VH-IVUS is good at assessing plaque composition and VH-IVUS–based plaque classification has been validated prospectively^5–7^; however, VH-IVUS has limited resolution such that thin fibrous caps cannot be directly imaged. In contrast, OCT provides high resolution to measure FCT, but has limited penetration. These complementary capabilities means that different combinations of OCT and VH-IVUS parameters may improve the ability to discriminate TCFA from other fibroatheroma (Table 5). Indeed, combining plaque burden ≥50% and LA_max_≥80° increased sensitivity to 86.4% and diagnostic accuracy to 80.5% for TCFA identification, with accuracy further increased to 85.0% if VH-defined fibroatheroma was also incorporated. However, the highest diagnostic accuracy (89.0%) was seen when VH-defined fibroatheroma was combined with FCT<85 μm for 3 continuous frames.

**Table 5.**
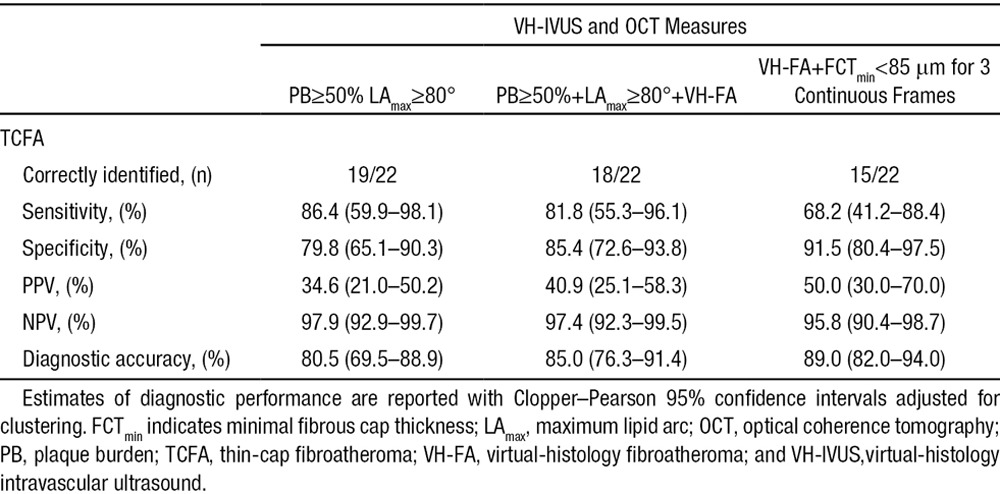
Combined VH-IVUS and OCT Imaging Plaque Features for Identification of Thin-Cap Fibroatheroma

## Discussion

Identification of advanced coronary plaques at risk of rupture remains a major challenge in cardiovascular research, with both VH-IVUS and OCT offering potential solutions. Although the accuracy of VH-IVUS for tissue characterization exceeds 93.5% in ex vivo studies,^3^ its diagnostic ability to identify TCFA is lower,^4^ and VH-IVUS studies consistently show a higher than anticipated prevalence of TCFA in vivo.^5,6^ In contrast, OCT has a higher sensitivity (90% to 92%) to identify lipid-rich plaques,^9^ but initial validation studies were often performed in noncoronary arterial territories with older, time-domain systems. Our study represents the first direct comparison of VH-IVUS and OCT alone and in combination for identification of advanced atherosclerotic lesions, compared with histology.

We show that both VH-IVUS and OCT can accurately quantify changes in tissue composition as plaques develop, including lipid/NC and calcification. Several IVUS and VH-IVUS features including plaque and NC areas were higher in TCFA versus other fiboratheroma, whereas only the number of continuous frames with FCT≤85 μm was higher in TCFA on OCT. Using existing definitions, both modalities could reliably identify both fibroatheroma and TCFA, with the diagnostic accuracy of OCT for TCFA identification marginally exceeding that of VH-IVUS (79.0% versus 76.5%). Importantly, we find that LA_mean_ or LA_max_ can identify TCFA, and propose a new cut off of LA_max_ ≥80° that may optimize TCFA detection. Finally, we show that combined VH-IVUS and OCT features of VH-defined fibroatheroma and FCT<85 μm over 3 consecutive frames markedly improved TCFA identification and led to a drop in the incidence of plaques classified as TCFA by imaging.

Our data highlight that both VH-IVUS and OCT can reliably identify fibroatheroma based on existing methods, with diagnostic accuracies exceeding 77.5%. Sensitivities were also reassuringly high (>70.1%), indicating that both modalities can readily identify large accumulations of NC/lipid, an observation supported by original validation studies assessing plaque tissue characterization.^3,9^ However, we also observed several false-positive results with both modalities, suggesting that TCFA may be overestimated in vivo. Our observations are supported by comparisons of clinical and histopathologic studies. For example, prospective imaging studies of nonculprit plaques have shown that VH-TCFA prevalence ranges from 21.6 to 60.3%^5,6^ with OCT-TCFA prevalence of ≈20%.^12^ In contrast, histopathologic studies show that ≈10% of all fibroatheroma are TCFA, representing 1.6% of the total coronary tree.^8^ Thus, future improvements toward in vivo TCFA identification should aim to reduce the number of false-positive observations.

Central to either fibroatheroma or TCFA identification is an objective assessment of overall lipid burden of a plaque and assessment of FCT. The limited tissue penetration of OCT has resulted in LA being suggested as a surrogate measure of plaque lipid area. However, it is unknown whether mean or maximal plaque LA should be used, or the threshold required for optimal TCFA identification. Clinical studies have used varying definitions with LA values between ≥90° and 180°.^11,12^ We find that LA_mean_ and LA_max_ are highly correlated and both measures produce high AUC statistics for fibroatheroma and TCFA identification. Using receiver-operating characteristic analysis, we find that LA_max_ of ≥80° provides optimum sensitivity to identify TCFA, compared with thresholds already in widespread use. Interestingly, we also observed that the number of continuous OCT frames with FCT<85 μm differed between TCFA and other fibroatheroma (*P*=0.03), with no observable difference in FCT_min_ (*P*=0.30). Indeed, we observed that 64.2% of all fibroatheroma had FCT_min_ <85 μm at some location within the plaque, implying that any single-frame measurement of FCT on OCT is unlikely to represent overall cap thickness. Quantification of the number of frames with FCT<85 μm in a plaque may be more robust measure of cap status as it mitigates against erroneous measures within single frames that could act to misclassify plaques as TCFA.^14^ These results should help to inform further studies aiming to discriminate TCFA from other plaque subtypes.

Our unique data set allows study of combinations of VH-IVUS and OCT imaging features to determine whether hybrid imaging could improve overall diagnostic accuracy for TCFA. We tested many combinations of VH-IVUS and OCT parameters and found that VH-FA and FCT<85 μm <3 consecutive frames improved diagnostic accuracy for TCFA to 89.0%, a level approaching clinical use. Although this combination will inevitably result in occasional false-positive observations, it seems significantly better than either imaging modality alone and suggests a role for hybrid imaging in atherosclerosis. Indeed, combined VH-IVUS and OCT has been previously performed ex vivo^15^ and in clinical studies,^16^ with both suggesting that imaging artifacts may be reduced through hybrid approaches. This position was further strengthened by recent autopsy data, highlighting that combined gray-scale IVUS and OCT can improve TCFA identification.^17^ Our study adds to this growing literature and seeks to overcome some of the limitations of previous autopsy work, where the histological prevalence of TCFA was low.^15,17^ Development of novel hybrid imaging catheters is ongoing, and it will be important to determine whether these devices improve our prediction of future cardiovascular events.

### Limitations

There are some limitations to our study. First, we examined ROI obtained from 14 hearts and our findings should be validated in larger data sets; however, this represented 258 coregistered ROI, resulting in robust statistical analysis. Second, coregistration between VH-IVUS and OCT is challenging and small longitudinal mismatches between imaging modalities may have a minor effect on overall results. However, an experienced imaging specialist performed all coregistration blinded to histological plaque classification, ensuring that the results presented are objective and free from bias. Third, 58 ROI could not be imaged with both modalities. However, all these ROI were located distally with few significant plaques, suggesting that overall values for accuracy are unlikely to be significantly affected. Fourth, histological processing can cause fragmentation and loss of plaque material, particularly in heavily calcified regions. Thus, the histological validation presented here may not precisely reflect the diagnostic performance of VH-IVUS and OCT in calcified vessels. Finally, these current results apply to VH-IVUS only and do not reflect the ability of other radiofrequency analysis methods to perform plaque classification.

### Conclusions

We demonstrate that both VH-IVUS and OCT can reliably identify advanced coronary plaques. We suggest refined cut-off values for TCFA identification for OCT, and find that combined VH-IVUS/OCT markedly improves overall diagnostic accuracy. Our results should assist clinicians and researchers in planning future studies to identify high-risk plaques in vivo.

## Acknowledgments

We thank Dr Ellen Moseley for her assistance in the preparation of histological samples. R.A. Parker was supported in this work by National Health Service (NHS) Lothian via the Edinburgh Health Services Research Unit. Finally, we thank the patients and staff at Papworth Hospital NHS Trust for their ongoing support.

## Sources of Funding

This study was funded by grants from the British Heart Foundation (BHF) (FS/13/33/30168), Heart Research UK (RG2638/14/16), the Cambridge National Institute of Health Research Biomedical Research Centre, and the BHF Cambridge Centre for Research Excellence.

## Disclosures

None.

## Supplementary Material

## CLINICAL PERSPECTIVE

Advanced atherosclerotic coronary plaques, including thin-cap fibroatheroma (TCFA), are thought to be responsible for the majority of myocardial infarctions. Thus, imaging modalities that can identify TCFA in vivo are of considerable importance. Virtual-histology intravascular ultrasound and optical coherence tomography (OCT) are invasive imaging modalities that can both assess coronary plaque morphology, allowing plaque classification. However, their ability to classify plaques has never been directly compared and it remains unknown whether combining these modalities would improve TCFA identification. Here, we performed ex vivo imaging of human coronary arteries, investigating whether virtual-histology intravascular ultrasound or OCT can perform accurate plaque classification, compared with “gold-standard” histology. We find that using existing imaging definitions, both modalities could reliably identify TCFA, with the diagnostic accuracy of OCT marginally exceeding that of virtual-histology intravascular ultrasound. In addition, we suggest refined cut-off values for lipid arc and fibrous cap thickness on OCT that may improve TCFA identification in vivo. Finally, we demonstrate that hybrid virtual-histology intravascular ultrasound/OCT imaging has potential to further improve TCFA identification. These results should hopefully assist clinicians and researchers in planning future studies to identify high-risk plaques.
