# Supplementary material for: Direct Comparison of Virtual-Histology Intravascular Ultrasound and Optical Coherence Tomography Imaging for Identification of Thin-Cap Fibroatheroma
Source: Circ Cardiovasc Imaging. 2015 Oct 20;8(10):e003487. doi: 10.1161/CIRCIMAGING.115.003487 (PMC4596008; doi:10.1161/CIRCIMAGING.115.003487)
Supplement: Supplementary file 1 [file hci-8-e003487-s001.pdf]

For the best experience, open this PDF portfolio in Acrobat X or Adobe Reader X, or later.

Get Adobe Reader Now!
