# Supplementary material for: Direct Comparison of Virtual-Histology Intravascular Ultrasound and Optical Coherence Tomography Imaging for Identification of Thin-Cap Fibroatheroma
Source: Circ Cardiovasc Imaging. 2015 Oct 20;8(10):e003487. doi: 10.1161/CIRCIMAGING.115.003487 (PMC4596008; doi:10.1161/CIRCIMAGING.115.003487)
Supplement: Supplementary file 7 [file hci-8-e003487-s007.pdf]

Supplementary Appendix

Direct comparison of virtual-histology intravascular ultrasound and optical coherence tomography for identification of thin-cap fibroatheroma

Adam J. Brown MD, Daniel R. Obaid MD, PhD, Charis Costopoulos MD, Richard A. Parker MSc, Patrick A. Calvert MD, PhD, Zhongzhao Teng PhD, Stephen P. Hoole MD, Nick E. J. West MD, Martin Goddard MD and Martin R. Bennett MD, PhD

Table of Contents

1. Supplemental Methods **3**

1.2 VH-IVUS plaque classification 3

1.3 OCT plaque classification 4

2. Supplemental Tables **5**

Table S1. VH-IVUS plaque classification inter-observer agreement 5

Table S2. OCT plaque classification inter-observer agreement 6

3. References **7**

Supplemental Methods

VH-IVUS plaque classification[1]

Image	Plaque Subtype	Description
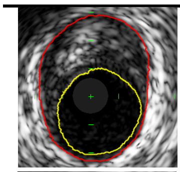	**Plaque**	Plaque burden (PB) >40% vessel cross-sectional area for 3 consecutive frames
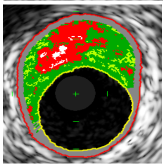	**Fibroatheroma** (VH-FA)	Plaque with >10% confluent necrotic core for 3 consecutive frames
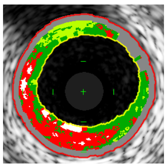	**Thin-capped fibroatheroma** (VH-TCFA)	VH-FA with confluent necrotic core in contact with the lumen for 3 consecutive frames
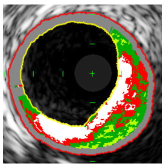	**Fibrocalcific plaque** (VH-FCa)	Plaque with >10% dense calcium for 3 consecutive frames
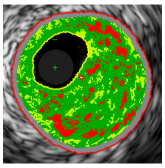	**Pathological intimal thickening** (VH-PIT)	Plaque not meeting either VH-FA or VH-FCa definitions

OCT plaque classification[2]

Image	Plaque Subtype	Description
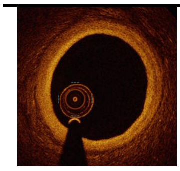	**Non-atherosclerotic vessel** (OCT-NAV)	Three-layered architecture of the vessel wall, with no or little evidence of intimal thickening
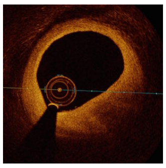	**Plaque**	Focal intimal thickening with loss of a layered structure to the vessel wall
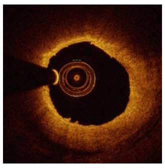	**Fibroatheroma** (OCT-FA)	Plaque with evidence of OCT defined lipid pool, where $LA_{Max} \geq 90°$
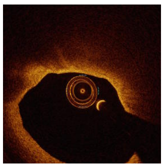	**Thin-capped fibroatheroma** (OCT-TCFA)	OCT-FA with $FCT_{Min} \leq 85\mu m$
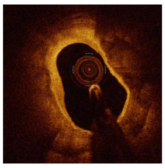	**Fibrocalcific plaque** (OCT-FCa)	Plaque with evidence of calcification, where any lipid-pool were $LA_{Max} \leq 90°$
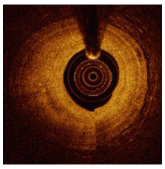	**Fibrous plaque** (OCT-Fib)	Plaque not meeting either OCT-FA or OCT-FCa definitions

Table S1 VH-IVUS plaque classification inter-observer agreement

Observer B \ Observer A	PB<40%	PIT	FCa	ThCFA	TCFA	**Total**
PB<40%	55	0	0	0	0	**55**
PIT	0	62	0	1	0	**63**
FCa	0	1	13	0	0	**14**
ThCFA	0	2	4	15	1	**22**
TCFA	0	2	9	5	42	**58**
**Total**	**55**	**67**	**26**	**21**	**43**	**212**

FCa, fibrocalcific; PB, plaque burden; PIT, pathological intimal thickening; ThCFA, thick-cap fibroatheroma; TCFA, thin-cap fibroatheroma

Table S2 OCT plaque classification inter-observer agreement

Observer B	Observer A: NAV	Fib	FCa	FA	**Total**
NAV	86	1	0	4	**91**
Fib	0	18	1	0	**19**
FCa	0	1	23	0	**24**
FA	1	4	4	65	**74**
**Total**	**87**	**24**	**28**	**69**	**208**

FA, fibroatheroma; FCa, fibrocalcific; Fib, fibrous; NAV, non-atherosclerotic vessel

References

1. Calvert PA, Obaid DR, O'Sullivan M, Shapiro LM, McNab D, Densem CG, Schofield PM, Braganza D, Clarke SC, Ray KK, West NE, Bennett MR. Association between IVUS findings and adverse outcomes in patients with coronary artery disease: The VIVA (VH-IVUS in vulnerable atherosclerosis) study. *JACC Cardiovascular Imaging*. 2011;4:894-901
2. Tearney GJ, Regar E, Akasaka T, Adriaenssens T, Barlis P, Bezerra HG, Bouma B, Bruining N, Cho JM, Chowdhary S, Costa MA, de Silva R, Dijkstra J, Di Mario C, Dudek D, Falk E, Feldman MD, Fitzgerald P, Garcia-Garcia HM, Gonzalo N, Granada JF, Guagliumi G, Holm NR, Honda Y, Ikeno F, Kawasaki M, Kochman J, Koltowski L, Kubo T, Kume T, Kyono H, Lam CC, Lamouche G, Lee DP, Leon MB, Maehara A, Manfrini O, Mintz GS, Mizuno K, Morel MA, Nadkarni S, Okura H, Otake H, Pietrasik A, Prati F, Raber L, Radu MD, Rieber J, Riga M, Rollins A, Rosenberg M, Sirbu V, Serruys PW, Shimada K, Shinke T, Shite J, Siegel E, Sonoda S, Suter M, Takarada S, Tanaka A, Terashima M, Thim T, Uemura S, Ughi GJ, van Beusekom HM, van der Steen AF, van Es GA, van Soest G, Virmani R, Waxman S, Weissman NJ, Weisz G. Consensus standards for acquisition, measurement, and reporting of intravascular optical coherence tomography studies: A report from the international working group for intravascular optical coherence tomography standardization and validation. *Journal of the American College of Cardiology*. 2012;59:1058-1072
