# Supplementary material for: Direct Comparison of Virtual-Histology Intravascular Ultrasound and Optical Coherence Tomography Imaging for Identification of Thin-Cap Fibroatheroma
Source: Circ Cardiovasc Imaging. 2015 Oct 20;8(10):e003487. doi: 10.1161/CIRCIMAGING.115.003487 (PMC4596008; doi:10.1161/CIRCIMAGING.115.003487)
Supplement: Supplementary file 8 [file hci-8-e003487-s008.docx]

**CLINICAL PERSPECTIVE**

Advanced atherosclerotic coronary plaques, including thin-cap fibroatheroma (TCFA), are thought to be responsible for the majority of myocardial infarctions. Thus, imaging modalities that can identify TCFA *in vivo* are of considerable importance. Virtual-histology intravascular ultrasound (VH-IVUS) and optical coherence tomography (OCT) are invasive imaging modalities that can both assess coronary plaque morphology, allowing plaque classification. However, their ability to classify plaques has never been directly compared and it remains unknown whether combining these modalities would improve TCFA identification. Here we performed *ex vivo* imaging of human coronary arteries, investigating whether VH-IVUS or OCT can perform accurate plaque classification, compared with 'gold-standard' histology. We find that using existing imaging definitions, both modalities could reliably identify TCFA, with the diagnostic accuracy of OCT marginally exceeding that of VH-IVUS. Additionally, we suggest refined cut-off values for lipid arc and fibrous cap thickness on OCT that may improve TCFA identification *in vivo*. Finally, we demonstrate that hybrid VH-IVUS/OCT imaging has potential to further improve TCFA identification. These results should hopefully assist clinicians and researchers in planning future studies to identify high-risk plaques.
